# An Analysis of Cellular Communication Network Factor Proteins as Candidate Mediators of Postpartum Psychosis Risk

**DOI:** 10.3389/fpsyt.2019.00876

**Published:** 2019-11-26

**Authors:** William Davies

**Affiliations:** ^1^MRC Centre for Neuropsychiatric Genetics and Genomics and Division of Psychological Medicine and Clinical Neurosciences, School of Medicine, Cardiff University, Cardiff, United Kingdom; ^2^School of Psychology, Cardiff University, Cardiff, United Kingdom; ^3^Neuroscience and Mental Health Research Institute, Cardiff University, Cardiff, United Kingdom

**Keywords:** brain, connective tissue growth factor, mood disorder, myelination, nephroblastoma-overexpressed

## Abstract

Postpartum (or puerperal) psychosis (PP) is a severe psychiatric condition associated with hallucinations, delusions, cognitive disorganization, and mood problems, which affects approximately 1–2 out of every 1,000 mothers shortly after childbirth. While the risk factors for, and co-morbidities of, PP are relatively well-defined, currently, the pathophysiology underlying the disorder is very poorly-specified. Here, I argue, on the basis of multiple lines of new evidence, that altered expression of the Cellular Communication Network (CCN) factor proteins (and of the heterodimerizing CCN2 and CCN3 proteins in particular), may be associated with, and possibly causal for, increased PP risk. Future preclinical and clinical studies should aim to test this hypothesis as empirical support for it would provide much-needed clues regarding the biological substrates of PP, and could point to predictive biomarkers for the condition.

## Postpartum Psychosis: an Introduction

Postpartum (or puerperal) psychosis (PP) is a severe psychiatric condition affecting approximately 1–2 of every 1,000 new mothers (1). The disorder is characterized by multiple symptoms occurring shortly after childbirth, often within the first few days; these can include hallucinations, delusions (often related to the baby), cognitive disorganization, and mood abnormalities including mania, depressive episodes, and extreme anxiety (2). PP can severely impact upon mother-child bonding and family dynamics, and is a leading cause of maternal death *via* suicide, and infanticide (3). Treatments for PP are relatively effective in many cases if administered promptly, and include a combination of antipsychotic, mood-stabilizing and sedative/anxiolytic drugs, and psychosocial support (2). However, some drugs lack efficacy, or exhibit unwanted side-effects, in some patients, and there are risks with administering some pharmacological treatments prophylactically during pregnancy due to teratogenicity issues (4). Thus, there remains a need for alternative treatments to be developed, which will, in turn, require an understanding of PP pathophysiology.

Our current understanding of PP molecular pathophysiology is extremely poor for a number of reasons, including the inaccessibility of the human brain, the low prevalence and high symptom heterogeneity of the condition, and a historic lack of relevant animal models (5). Clinical studies have tended to focus upon obvious candidate biological systems in the perinatal period, including variations in steroid hormone levels, immune system fluctuations, and genes influencing the dopaminergic, serotonergic, and stress-response systems. While there is some evidence for altered sensitivity to postpartum steroid hormone levels, immune system hyperactivation, and a nominally-increased frequency of specific genetic variants within serotonergic genes in patients with PP, results from clinical studies so far have been limited and inconsistent (5). The nature of PP limits our ability to perform robust, hypothesis-free, large-scale biochemical, and genetic analyses, including genome-wide analyses which are now beginning to bear fruit in helping us to understand the biological basis of other, more common, mood, and psychotic illnesses (6); even with ongoing multi-national collaborative efforts, the sample sizes required to identify a reasonable proportion of genetic risk variants (and thereafter risk pathways) are unlikely to be achieved in the near future. Hence, for rare disorders such as PP, investigations of sensible candidate biological pathways may still be warranted.

Ideally, we would like to know during pregnancy, or even before pregnancy, which women are at increased risk of being affected by PP, so that they can be closely monitored and receive early intervention for maximum therapeutic benefit. While we know that women with a previous history of PP, bipolar disorder or schizophrenia are at substantially increased risk of PP, approximately half of women who experience PP have no prior psychiatric history (7). Biological differences between women experiencing PP, and those who do not, could potentially represent predictive biomarkers for the condition.

In this perspective, I hypothesize, based upon several lines of converging preclinical and clinical evidence, that the expression of Cellular Communication Network (CCN) family member proteins may be disrupted in women at risk of PP, and that detailed analysis of this system in future studies could feasibly yield insights into the pathophysiology of PP and signpost predictive biomarkers.

## The Cellular Communication Network (CCN) Factor Gene Family

The CCN gene family comprises six members formerly known as cysteine-rich angiogenic inducer 61 (CYR61, now CCN1), connective tissue growth factor (CTGF/CCN2), nephroblastoma-overexpressed (NOV/CCN3), WNT1-inducible signaling pathway 1 (WISP1/CCN4), WNT1-inducible signaling pathway 2 (WISP2/CCN5), and WNT1-inducible signaling pathway 3 (WISP3/CCN6). The family member proteins share a tetramodular domain and regulate a variety of developmental and physiological processes including cell adhesion, migration, proliferation, differentiation, and survival (8). Although CCN proteins are expressed intracellularly, they appear to play a particularly important role within the extracellular matrix where they interact with multiple components, including a variety of cell membrane receptors (8); this site of action is pertinent as extracellular matrix proteins have been highlighted as candidate modulators of mood disorders (9). The CT domain shared across CCN members is a common motif in dimerizing proteins (10), and there is evidence that CCN members such as CCN2 and CCN3 can form homo or heterodimers; these two proteins may exert opposing effects (11), and in the nervous system, this is exemplified by CCN2 and CCN3’s anti- and pro-myelination effects, respectively (12–14). Below, I argue that six key features of the CCN family member proteins (and specifically CCN2 and CCN3), render them plausible biological mediators of PP risk.

### Altered Gene Expression in Animal Models Displaying Abnormal Maternal Behavior

It should initially be acknowledged that complex psychiatric conditions such as psychosis are challenging to model accurately or comprehensively in animals (15). This caveat notwithstanding, recent work in three independent, and distinct, animal models demonstrating abnormal maternal behavioural phenotypes has indicated correlated CCN2 and CCN3 brain gene expression changes.

In a pharmacological mouse model, the steroid sulfatase (STS) enzyme was acutely inhibited in new mothers to mimic postpartum steroid sulfatase deficiency which is associated with increased postpartum psychopathology risk (16) [including potentially increased postpartum psychosis risk (17)] in women. Mouse mothers in which STS was inhibited exhibited apparently normal dam-pup interactions, but altered anxiety-related behaviors and a reduced startle response; the specific pattern of anxiety-related behaviors implicated an underlying genetic substrate within a small region of chromosome 15 (18). Screening of the 17 genes within this interval identified CCN3 as the only significantly differentially expressed (upregulated) gene in the brain between vehicle and inhibitor-treated mothers, and follow-up analyses of other CCN family members demonstrated significant brain overexpression of CCN2 and CCN4 genes in the latter group (18).

In a genetic mouse model, the postpartum behavior of wild-type mothers bearing transgenic pups overexpressing the imprinted gene *Phlda2* was compared to that of wild-type mothers bearing wild-type pups (19). After birth, wild-type dams exposed *in utero* to transgenic offspring exhibited decreased nursing and grooming of pups, and an increased focus on nest building, relative to wild-type mothers exposed to wild-type pups. Analysis of global gene expression in the hippocampus and hypothalamus of mothers bearing transgenic pups revealed several significant differences from that in mothers bearing wild-type pups, including substantial upregulation of hippocampal *Ccn3* expression (>2.1-fold change) (19).

In pigs, a small proportion of new mothers demonstrate aggression towards their offspring, in some cases killing one or all of the litter. The “infantidal sow” was the first animal model proposed for PP, and exhibits some degree of face validity for the condition: the extent of aggression is influenced by familial, hormonal, and environmental factors, is greater for first litters, and is associated with behavioral correlates such as anxiety and restlessness (20). A preliminary analysis employing RNA sequencing to compare hypothalamic gene expression in infanticidal and non-infanticidal sows revealed increased CCN2 (CTGF) expression (∼1.3-fold) in the former group as one of the top 10 largest expression differences (21). Genetic analysis of the pig model has also implicated *PAX3* in postpartum aggression (22); the chromosomal translocation-derived fusion transcription factor PAX3-FKHR appears to act as a transcriptional activator at *CCN3* (23).

### Location Under a Genetic Linkage Peak for PP

A seminal genetic study in a population of women with bipolar disorder who developed PP identified a genome-wide significant linkage signal on chromosome 16p13, and a genome-wide suggestive linkage signal at 8q24 (24). The suggestive linkage signal spanned 14 cm, from 135.7–149.7 cm; the *CCN3* gene is located at 138 cm, and as such could potentially fully, or partially, explain this signal.

### PP-Relevant Spatiotemporal Gene Expression Patterns and Influence on Relevant Behavioral Phenotypes

CCN genes are expressed in the developing and adult mammalian brain, with associated proteins apparently more highly expressed in neurons than in glia (25). Across species, the highest expression of CCN2 and CCN3 is seen in brain regions underpinning higher cognitive functions and emotionality, including the hippocampus (CA1 region), and frontal and temporal cortices (25–27); interestingly, these brain regions appear to be structurally distinct in women at risk of PP who develop the condition relative to those who don’t (28), while alterations in the structure and function of the CA1 region of the hippocampus are among the most replicated neuroimaging findings in individuals with psychotic and/or bipolar illness (29).

**Figure 1:**
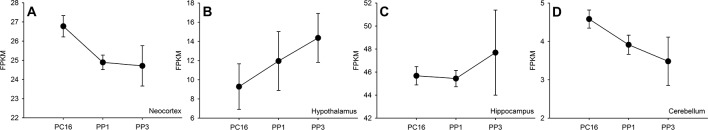
Expression of *Ccn3* across neocortex **(A)**, hypothalamus **(B)**, hippocampus **(C)** and cerebellum **(D)** regions of C57BL/6J mouse brain from mid-late pregnancy (postconception day 16, PC16) to postpartum days 1 and 3 (PP1 and PP3). RNA sequencing data are taken from Ray et al. (30) and expression is measured via Fragments Per Kilobase of transcript per Million mapped reads (FPKM) across three biological replicates per timepoint (mean and standard error of the mean shown for each timepoint).

*A priori*, one might expect the expression of candidate genes for PP to fluctuate within the mother’s brain between late pregnancy and into the postpartum period. Although information on the brain expression of CCN genes across these timepoints in humans is inevitably lacking, there is some evidence from rodents that maternal brain CCN expression does change in a spatiotemporally-specific manner from mid-late gestation and into the postpartum period (30). Notably, CCN3 expression decreases in both the maternal neocortex and cerebellum across these timepoints, but increases significantly in the hypothalamus (**Figure 1**); CCN2 levels, in contrast, remain relatively stable across all four brain regions.

If there is a true link between abnormal CCN family gene expression and PP, we might reasonably expect to see altered expression of these genes/proteins in tissue samples from individuals with psychotic illness. In a recent comparison of global gene expression in prefrontal parvalbumin cells from individuals with schizophrenia versus healthy controls, more than 800 transcripts were identified as being differentially expressed, with CCN3 being one of the top hits (>40% change in expression) (31). Second, an analysis of plasma analytes in individuals at high risk of developing psychosis has suggested that conversion may be mediated by CCN2 (CTGF) (32). Thirdly, CCN3 (NOV) is functionally related to genes in which mutations are disproportionately highly observed in schizophrenia cases relative to controls (33). Finally, the protein discoidin domain receptor 1 (DDR1) has been proposed as a receptor for CCN3 (34); DDR1 is involved in myelination processes in the brain (35), and there is robust evidence for an association between genetic variants around the *DDR1* locus and psychotic illness, possibly mediated *via* white matter abnormalities (36–39). Conceivably, therefore, any CCN3 effects on psychosis risk might be mediated *via* DDR1.

In addition to effects on psychosis risk, perturbed expression of CCN2 and/or CCN3 might impact upon mood symptoms and relevant personality traits. CCN2 (CTGF) has recently been implicated as a prodepressant molecule on the basis of human and animal model studies (40), while CCN3 hippocampal expression is decreased ∼1.7-fold in the “chronic social defeat” mouse model of depression (41). Consistent with the results from the STS enzyme inhibition study, brain-specific deletion of *Ccn3* has an anxiolytic effect in mice (42). New findings from a large Mendelian Randomization study have suggested that plasma CCN2 is significantly causally-associated with neuroticism and possibly also with depression (p = 0.07), and that plasma CCN3 is significantly causally-associated with anxiety/panic attacks and agreeableness (43, 44); in bipolar disorder, neurotic and (negative) agreeableness traits are reliable predictors of depressive and manic symptoms, respectively (45).

Among some women with bipolar disorder, sleep deprivation appears to be a trigger for acute mood (manic) symptoms, and it is these individuals who appear to be at particularly high risk of developing PP (46). Emerging data hints that elevated blood CCN3 levels are associated with obstructive sleep apnea (OSA), a common condition associated with impaired sleep and subsequent cognitive processing, and with irritable or depressed mood (47). Feasibly, a predisposition to sleep disruption as a consequence of high CCN3 levels and heightened OSA risk may play a role in vulnerability to PP.

### Expression Regulated by Pro- and Anti-Psychotic Factors Relevant to PP

There is evidence from both *in vivo* and *in vitro* studies that exposure to pro- and anti-psychotic factors can influence CCN family gene expression; however, many of these studies have focussed on tissues other than the brain, and the extent to which their results can be generalized to this tissue remains to be confirmed.

Of relevance to the idea that a precipitous drop in maternal circulating steroid hormones (notably oestrogens) may somehow elicit psychosis in the postpartum period, work in rats has demonstrated that ovariectomy, and the associated decrease in circulating oestrogens, results in significantly decreased *Ccn3* (*Nov*) expression in the hippocampus (48). Chronic stress results in upregulation of the *Ccn2* (*Ctgf*) gene in mouse hippocampus (49), while the administration of psychotomimetic or antipsychotic drugs results in increased CCN gene expression across the rodent brain (50, 51). In the STS inhibition mouse model described above, the elevation in brain *Ccn3* expression could be alleviated by concurrent administration of the clinically-efficacious antipsychotic ziprasidone (18), while in rats, oral administration of lithium (a mood-stabilizing drug used to treat PP), is associated with substantial, and relatively specific, upregulation of *Ccn3* kidney expression (52). In women with a history of bipolar disorder/psychosis, smoking appears to be associated with vulnerability to psychotic episodes during pregnancy and in the puerperium (53, 54); in female mouse lung tissue, extended exposure to cigarette smoke has been associated with downregulation of CCNs 1, 3 and 4 (55).

Finally, *in vitro* studies have shown that CCN expression can be finely regulated by a diverse range of inflammatory mediators including cytokines (TNFα, IL1β, TGF-β), prostaglandins, nitric oxide, histamine, serotonin, and extracellular matrix enzymes in a tissue-specific manner (56).

### Role as a Nexus Between PP-Relevant Immune System and Neuroanatomical Measures

Individuals with first-onset PP have been reported to show hyperactivation of the monocyte/macrophage arm of the immune system (57), possibly as a downstream consequence of changes in the abundance and/or activity of subsets of immunosuppressive regulatory T-cells (Tregs) (57, 58). The proportion of circulating Tregs also appears to be abnormal (generally lowered) in individuals with bipolar disorder (59–61).

Although little is currently known about the neuroanatomy of PP, pilot data indicates that individuals at high risk of PP exhibit abnormal myelination in the temporal lobe, and in sublobar areas (62), and one case of PP presented with white matter abnormalities within the splenium of the corpus callosum (63). Interestingly, in patients with bipolar disorder, the frequency of circulating Tregs correlates positively with markers of white matter integrity (64). Together these results suggest: a) that defective myelination processes may predispose to PP and associated conditions, and b) the possibility of a biological link between immune function and myelination processes across these conditions.

Work by Dombrowki and colleagues has shown that CCN3 is secreted by Tregs, and mediates brain (re)myelination processes in mammals (12). On the basis of this finding, we have proposed that perturbations in the Treg-CCN3-(re)myelination axis may confer vulnerability to PP (65). The high expression of CCN3 in the temporal cortex, and adjacent structures, may potentially explain the myelination abnormalities seen here in patients with PP. Additionally, an *in vitro* study in mouse cortical neurons in which CCN3 was overexpressed showed impaired midline crossing of callosal projection neurons (66), providing a viable explanation for the corpus callosum structural abnormalities described in the PP case above.

### Putative Role in Conditions Comorbid With PP

Convergent findings from large general population, and disease-specific cohorts, support a robust association between the medical condition pre-eclampsia and postpartum psychiatric symptoms (67, 68). Pre-eclampsia is a potentially life-threatening complication of late pregnancy, characterized by the onset of high blood pressure and proteinuria (69). Like PP, the pathophysiology of pre-eclampsia is poorly-understood, but abnormal placental invasion and vascularization processes are thought to be important (69); chronic maternal hypertension is a significant risk factor (69, 70).

Biological factors which have pleiotropic (independent) effects on both pre-eclampsia and postpartum mood disorder risk may explain the observed link between the two conditions. Alternatively, these factors may solely (or predominantly) influence pre-eclampsia risk, and the development of this condition may then precipitate symptoms associated with PP *via* mechanisms such as heightened perinatal stress or *via* adverse effects of hypertension on neural processes. CCN proteins, and CCN3 in particular, represent plausible pleiotropic biological risk factors.

CCN family members influence angiogenesis across a variety of tissues, and in the placenta CCN1 and CCN3 are expressed in the endothelial cells of placental vessels with expression increasing throughout pregnancy (71); in the placenta, CCNs1-3 seem to act as regulators of trophoblast proliferation and migration, with CCN2 acting antagonistically (71). CCN1 and CCN3 levels are decreased in both the placental tissue and serum of mothers with early-onset pre-eclampsia, whereas CCN2 levels are increased in the serum of women with severe pre-eclampsia (71). In addition to a possible role in pre-eclampsia pathophysiology, CCN3 may play a role in affecting blood pressure more generally; the gene is very highly expressed in the zona glomerulosa of the adrenal gland, a tissue involved in the regulation of blood pressure *via* aldosterone secretion (72). Consistent with this, genomic studies have identified a genetic polymorphism within *CCN3* (rs2071518) associated with blood pressure regulation (73), and plasma CCN3 is highly-significantly causally-linked to blood pressure (43, 44).

Given the well-established relationship between bipolar disorder and PP risk, one might expect CCN proteins to influence cellular processes relevant to both disorders. CCN3 is thought to regulate intracellular calcium levels (74), and aberrant calcium signaling has been implicated in both bipolar disorder (75) and PP (76) risk. Finally, CCN proteins can influence Notch and Wnt signaling cascades (10, 71), the dysfunction of which has been reported in both bipolar disorder and schizophrenia (77, 78) and in cases presenting with postpartum psychiatric symptoms (79).

## Discussion

**Figure 2:**
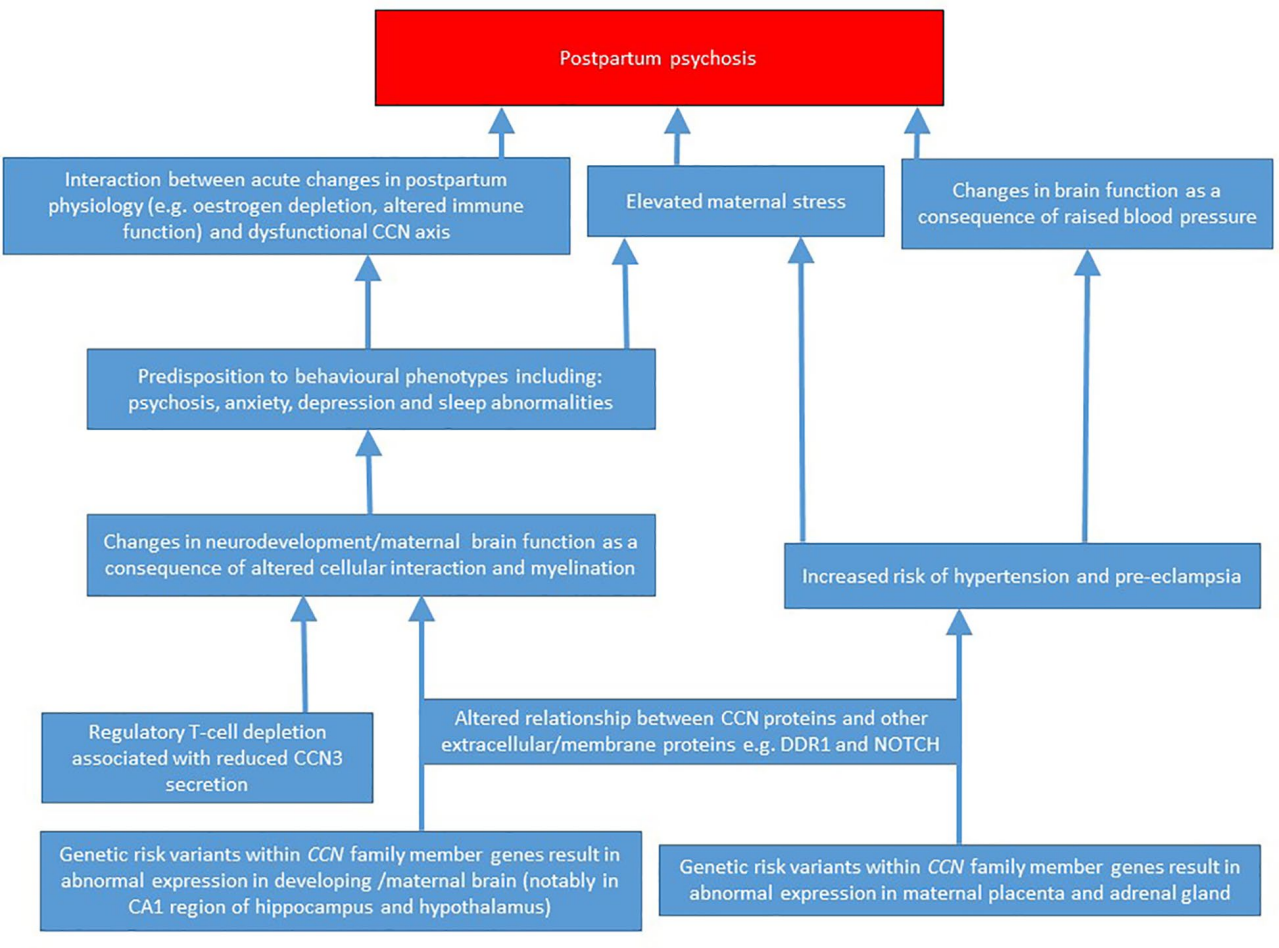
A putative pathophysiological model for how abnormal CCN family member expression may predispose to postpartum psychosis.

Currently, the pathophysiology of PP is poorly-understood. The rarity and complexity of the condition precludes large case-control biochemical and genetic studies, and there is arguably a need to focus on candidate systems. Historically, however, candidate gene or system-led approaches have been associated with high rates of false positive and negative findings (80), though some of this poor reproducibility may be accounted for by the relatively arbitrary selection of candidates based upon limited and unreliable theory. Above, I have discussed why I believe the CCN family represents a strong candidate system warranting further investigation in PP; a putative pathophysiological model based upon these arguments is presented in **Figure 2**. Despite there being a strong relationship between a prior history of bipolar disorder and PP risk, to date, there is little evidence from genomic and physiological studies for abnormal CCN function in bipolar disorder; this “absence of evidence” may be due to technical limitations, or alternatively, as CCN gene/protein levels fluctuate considerably throughout pregnancy and the postpartum period, it is plausible that it is specifically abnormalities in the expression/function of these proteins (against a background of bipolar disorder-associated pathophysiological changes) that confer PP risk. Additionally, it should be appreciated that while a link between CCN proteins and PP is explicitly discussed here, the functions/attributes of the CCN family mean that these proteins could also feasibly play a role in disorders with overlapping symptoms and biology, notably postpartum depression and anxiety.

Future work may involve comparing the levels of CCN2 and CCN3 proteins (and their interactors) in peripheral tissues in individuals with PP, in individuals at risk of PP, and in healthy controls across pregnancy and the postpartum period, as well as comparing the sequences of these genes across groups. Parallel animal model studies, in which levels of CCN2 and CCN3 proteins are systematically varied and PP-relevant neurobiological and behavioral measures assessed, may be useful for indicating causality.

Should the work above suggest that changes in the CCN family are associated with, or causal for, an increased risk of postpartum psychopathology, they could act as predictive biomarkers, and may be amenable to normalization through therapeutic approaches currently under development (81).

## Author Contributions

The author confirms being the sole contributor of this work and has approved it for publication.

## Funding

The work was supported by Medical Research Council (MRC) United Kingdom Centre Grant MR/L010305/1 (https://mrc.ukri.org/) and Cardiff University. The funders had no role in study design, data collection and analysis, decision to publish, or preparation of the manuscript.

## Conflict of Interest

The author declares that the research was conducted in the absence of any commercial or financial relationships that could be construed as a potential conflict of interest.
